# A Demonstration of Medical Cases

**Published:** 1909-06-12

**Authors:** William Gowers

**Affiliations:** Physician to the National Hospital for the Paralysed and Epileptic, Queen Square, London, and Consulting Physician to University College Hospital


### Hospital Clinics.

### A DEMONSTRATION OF MEDICAL CASES.

By Sir WILLIAM GOWERS, M.D., F.R.S., F.R.C.P.; Physician to the National Hospital for the Paralysed and Epileptic, Queen Square, London, and Consulting Physician to University College Hospital.

(A Lecture delivered at the Polyclinic, May 4, 1909.)

### A Doubtful Case of Lateral Sclerosis.

Gentlemen, you will notice that this woman is just able to walk, but she does so with difficulty. Her age is 66, so she has reached the degenerative period of life. She suffered from nervous symptoms 13 years ago, brought on by her enforced separation from her husband after 25 years of married life. The separation brought on attacks of unconsciousness, the attacks sometimes lasting two days. She has not been able to walk properly since that date. She has not had an attack of unconsciousness for over five years.

Her heart, lungs, and kidneys are quite healthy. She is not aware of having had any convulsions; she says that in the attacks I have just mentioned she felt very queer and went off. Apparently they were not epilepsy. Her knee-jerks are said to be present during these attacks, but very feeble. There is anæsthesia of both legs, up to near the body, and of the left arm. Her left hand, as you see, is in a curious contracted condition; and the contraction of the flexors very firm. The fingers of the right hand are quite supple, and she can feel in that hand. The question is whether the condition is functional, or organic in nature.

I think there must be organic changes on the left side. Both knee-jerks are vigorous, and there is a very quick response. When I raise the right foot the left leg moves off the ground, and that is always a very important symptom, which shows organic disease. It is due to the tendency to contraction of the muscles uniting both legs to the pelvis. There is great rigidity, and neither foot can be flexed to a right angle. I should certainly say this is organic disease; that she has lateral sclerosis of both sides, involving the arm on the left side.

I remember being asked, some years ago, to see a case at another hospital about which there was great difference of opinion. The patient in this instance was a young woman, who had become paraplegic when she was walking near the hospital, and was brought in. She had had some mental trouble. Of course, mental trouble is regarded as a frequent cause of functional disease, but it does not exclude organic disease. The patient was lying in bed, and I took hold of one foot and raised it up, and the other leg moved with it; and I said at once that it was organic disease, that there was lateral sclerosis, probably secondary to an acute lesion of the dorsal cord: and so it proved on further examination. It is a symptom which is revealed at once, on the first examination of the patient.

This patient's sphincters are unaffected. You often see cases of spastic paraplegia due to lateral sclerosis, in which both legs have become affected, and after the legs, one arm. Sometimes it involves all four limbs, but it more frequently ceases when one arm has become involved, and I think this is a case of that character.

With regard to the treatment of this patient she ought not to be forced to do anything, but ought to have upward massage to the legs and arms. No faradism, which rather excites a greater degree of contraction; and something ought to be put inside the fingers, such as an indiarubber air-ball, to exert pressure gradually outwards. That may gradually produce more relaxation and flaccidity. She has no pain. Of course, functional disease is common, but it does not follow that all cases of disease in women are functional, and we have always to beware of coming to such a conclusion.

### A Diagnosis of Post-Hemiplegic Chorea.

Here is a man æt. 42, whose trouble started some years ago with a quarrel with his wife. Although there was subsequent reconciliation there was great emotional disturbance at the time. Six or seven months afterwards he had what he describes as a stroke, starting with numbness all up the right side. He walked for 15 minutes after the commencement of the seizure, but could not then walk any more, and was taken to Middlesex Hospital in a cab. He remained there seven weeks, and was unable to walk during his stay. He has continued to have this involuntary movement of the arm ever since.

His age was 40 when it came on. He says he has never had any venereal disease, but you hear him admit he has been lucky, which means he has been exposed to the risk of it, and therefore you cannot exclude it. It is the least offensive way of ascertaining the possibility of it. He says he has never had gonorrhœa. He married at 18 years of age.

Hemiplegia at the age of 40, if it is organic, suggests arterial disease. But he has not reached the period of arterial degeneration; therefore, if there is no cause of embolism, the arterial disease is probably syphilitic. His description is like that of the onset of organic hemiplegia, of a lesion in the posterior part of the internal capsule of the brain, near the region for the sensory symptoms, and so the onset is attended by a disturbance of sensory functions. He describes it as numbness running up the side, and down the arm. The spasm is a little like that which you see in functional cases. There is a constant spasm, not exactly a tremor, but a somewhat irregular contraction.

The symptom which would suggest simple functional disorder is that which Dr. Harry Campbell mentioned, that he had anæsthesia in the hand some time ago, and now it is gone. The feeling, however, has just lately come back. He cannot make any definite use of the hand. There is ankle clonus, but he has had no dorsi-flexion from the plantar reflex. My impression is that it is an organic case, possibly with some functional symptoms superadded. It might be called post-hemiplegic chorea.

### Functional Spasm.

This boy, æt. 17, has spasm of the head, as you see, which he has had nine years. He has a small head; he is very musical, and plays the piano very well. During his playing the head does not jerk very much. These shakings are apparently functional—that is to say, they do not depend upon any organic lesion. But they may persist so long that we must consider there are nutritional changes in the nerve elements. In the absence of voluntary movement the spasm is very slight.

Cases of this description are essentially different in nature from chorea, but their precise nature is not known. There should be as much rest as possible: that is the most important elementary treatment, with fresh air and massage. Hypnotism may also be tried. Of drugs, I think arsenic and hyoscine are most likely to do good.

### Tabes and Syphilis.

This man, æt. 46, has evidence of tabes. He had syphilis 25 years ago. His symptoms have been bad two years, but he first got occasional pains about six years ago—that is to say, 19 years after the infection. His sight is not bad, but if he raises the left eyelid he sees double. He has extremely small pupils. There is no action to light, but a distinct reaction when he makes an effort at accommodation. That is the characteristic condition of pupils in locomotor ataxy—in other words, the Argyll-Robertson pupil.

As you know, those who have had tabes have had syphilis, and this pupil-symptom is a post-syphilitic symptom; it is not a tabetic symptom; you often see it without any tabes, but I think you never see it except in those who have had syphilis. So it is a symptom which is of very great practical importance. Tabes is usually preceded by syphilis, and if you find this condition of pupil you may dispense with the inquiry as to whether the patient has had syphilis. Of course, if it is absent, that is not of the same significance as to there not having been syphilis, as if it were present. This man has been married 17 years. The relation of the disease to syphilis is a matter which is simply one of inference. Tabes is not a syphilitic disease, although it is a constant consequence of syphilis; it is not a disease which can be cured by the means which are so effective in removing the true lesions of syphilis.

The best hypothesis which can reasonably be formed is that the organisms of syphilis leave a toxic agent in the body, which, without any fresh infection from increase in the organisms, develops without any fresh symptoms of true syphilis, and goes on increasing, although not inevitably, but with certainty so far as most of the symptoms it produces are concerned. The lesions of tabes which have been produced seldom pass away, and they vary very much in their nature and character. As you know, in most cases not only is the reflex of the iris lost, but the knee-jerk is lost—there is not the least response. I saw a man a few days ago who had been said by two physicians in London, who are extremely good men, to have locomotor ataxy. He had had syphilis long before. A very well-known physician in Edinburgh said he had nothing of the kind. When his knee-jerks were tried there was the usual response, there was contraction in the rectus femoris, moving the leg; but on noticing it carefully there was always a short but quite definite interval between the tap and the response. I could not tell you what the length of that interval was, but it was definite, and was present whenever an attempt was made to elicit the jerk. That is not a true jerk; it is a true reflex action from stimulation of the skin or muscle nerves, and always requires a certain time for its production. The true knee-jerk occurs instantly; it is due to a state of irritability of the muscle fibres to the local impulse given by tapping the tendon. I have no doubt that the man I spoke of has true tabes, although he had been supposed by a good authority not to have. This man has pains, and they are a very frequent symptom, but it is astonishing in what variety they occur, the amount of the pain which is felt, and the course which it takes.

### The Pains of Tabes.

Sometimes these patients have pains for a time, and then they cease. What the precise cause of the pains is it is not easy to say with confidence. Some believe they are due to changes within the spinal cord, but I think it is more likely that they are due to the degenerative changes in the extremities of the fibres, of which we have abundant evidence. The seat of the pains varies. Sometimes they are deep; sometimes apparently in the cutaneous nerves. Pains may persist in one part for 24 hours, and then depart for weeks, or even months; and in the meantime there is perhaps another attack at another spot. This man tells us the pain is like that produced by hot wire and hot water. The temperature pain is a not very common tabetic symptom, but when it does occur it is often severe. This man's pains seem to be deep, and those are less easily relieved than are the pains which seem to be situated in the skin.

Internal agents do them good, especially aluminium, given for a considerable time, or salicylate of soda, but the immediate relief of the pain, unless it is superficial, is much less easy. Superficial pain can often be relieved by an injection of cocaine in the upper region of the part where it is felt. What is better than the injection of cocaine is to administer the cocaine by means of a voltaic battery, by saturating the positive pole with cocaine, and the negative with salt and water, putting the positive pole on the region where the pain is felt, and the negative pole—anywhere. The current goes in at the positive pole and comes out at the negative, and it tends to carry the cocaine with it into the skin. You may render a patch on the hand insensitive in ten minutes by applying cocaine in this way. It is curious how a tabetic affection of the nervous system seems generally to be limited to some nerves, affecting other nerves very little.

This man's deeper nerves seem to suffer much from the irritation, caused by the very slow minute changes going on in them in consequence of the degeneration, and yet the nerves of the muscles themselves can be very little affected, or his gait would be much more ataxic. His sexual power is not lost, but is much diminished. He has no difficulty in passing his water, and his bowels are regular. This man illustrates the general affection of tabes. He has had much iodide, apparently without benefit. He has no cough or gastric crises. Touch is generally not impaired, and pain is much impaired, and to test touch, nothing is better than the feather of a quill pen, the other end being cut into two separate points, to test sensibility to pain, they seldom draw blood in the examination as does a pin.

### A Typical Case of Pseudo-Hypertrophic Paralysis.

Here is a little boy whom I happened to see at the hospital yesterday. The case is an extremely typical example of pseudo-hypertrophic paralysis. He seems to be the only case of the kind in his family. He is not a very bright boy; in this he is an exception, for in most cases of pseudo-hypertrophic paralysis the mental development is good. These patients are not able to take the ordinary amount of physical exertion, and so their minds often get a little over-cultivated. There is nothing else special about his aspect.

I imagine that you all know the general characters of pseudo-hypertrophic paralysis, and most of you must have seen cases. You know the common features of the disease, and this boy shows some which are very interesting. He has conspicuous enlargement of the calves, and some enlargement of the thighs, but not just above the knees; so the calves are an inch larger in circumference than the minimum of the thighs. When he sits his back is straight; the common curvature comes on when he stands. The disease depends on deficient growth of muscular fibres and over growth of the interstitial tissue and fat. As I pointed out many years ago, the infra-spinatus in these cases is generally enlarged, just as the calf muscles are. You see here the prominence formed by the infra-spinatus, but the lower part of the pectoralis is small, and the latissimus dorsi is also small; they are indeed absent, and it seems probable they were congenitally absent. The serratus magnus is also much affected. That is why the apex of the scapula projects forwards as he holds his arm out. He also shows very typically the characteristic way of getting up from the floor, putting his hands on the lower part of the femora to raise himself. Ordinarily the knee is extended by the extensors in the thigh, but the weight to be raised is at the upper extremity of the femur. And the muscle which extends the knee acts from, on the average, the middle of the femur, so that the power is between the fulcrum at the femur and the weight at the top of the femur. That is the position in which the power acts to least advantage. Whereas the boy, by putting his hand on the lower part of the femur, transfers for the moment much of the weight of the trunk to the lower part of the femur, close to the knee. It is curious that that is so seldom seen in any other affection, probably because this affection develops during the period of growth, and in the body of the young child this contrivance is readily adopted. Its importance as a diagnostic sign is great.

### Tremor in Paralysis Agitans.

This man's age is 46, and he has paralysis agitans. You will have noticed with what short steps he came in, and that his right arm shakes. I place a piece of paper on the floor and ask him to step on it as he walks, and you see that by such a simple device he feels impelled to take longer steps, and his gait is really quite altered by it. His arm began to shake when he was 38, which is a very early age for paralysis agitans to commence. The condition begins most frequently between the 50th and 65th years. In his hands there is a little tendency to the contracture of the interossei, and there is spontaneous tremor. The left arm does not shake so much. The tremor is a little increased by effort. But, for diagnostic purposes, a more significant fact is the expressionless aspect of the face, and the tendency to fixation of the limbs. The knee-jerk is usually normal, not increased.

The diagnosis between tremor due to other causes and paralysis agitans is often very difficult; but when you find in addition general fixation of the muscles, you can have no doubt about the diagnosis. He has a tendency to dislike heat, and he likes as few bedclothes as possible—a common tendency in paralysis agitans. Some dislike cold even more than they object to heat. He has some little tremor of the tongue, but not much.

